# Oral health, psychosocial aspects, and subjective happiness among Brazilian Army recruits: a pathway analysis

**DOI:** 10.1590/1807-3107bor-2026.vol40.025

**Published:** 2026-05-18

**Authors:** Maria Laura Castro Alves Ribeiro Gazola, Jessica Klöckner Knorst, Nicássia Cioquetta Lock, Patrícia Kolling Marquezan, Julio Eduardo do Amaral Zenkner, Luana Severo Alves

**Affiliations:** (a) Universidade Federal de Santa Maria – UFSM, Department of Restorative Dentistry, Santa Maria, RS, Brazil; (b) Universidade Federal de Santa Maria – UFSM, Department of Stomatology, Santa Maria, RS, Brazil; (c) Universidade Federal de Santa Maria – UFSM, Department of Microbiology and Parasitology, Santa Maria, RS, Brazil

**Keywords:** Happiness, Adolescent, Cross-sectional Studies, Oral Health, Quality of Life

## Abstract

This study investigated socioeconomic, psychosocial, behavioral, and clinical factors directly and indirectly associated with subjective happiness among recruits from southern Brazil. This cross-sectional study included a sample of recruits performing mandatory military service at two military bases in southern Brazil. Questionnaires were administered to collect socioeconomic data (mother's education, family income, and participant education level), behavioral habits (toothbrushing frequency and flossing), and psychosocial variables, such as sense of coherence (SoC-13 scale), oral health-related quality of life (OHRQoL), assessed using the Oral Health Impact Profile (OHIP-14), and degree of happiness (Subjective Happiness Scale). A clinical dental examination was performed by a single trained and calibrated examiner to determine the gingival bleeding index and dental caries experience (DMFT, number of decayed, missing due to caries, or filled permanent teeth). Pathway analysis was conducted to test the direct and indirect paths linking socioeconomic, psychosocial, behavioral, and clinical factors to happiness (Stata software). Results are presented as beta coefficients (β) and *p* values. A total of 499 recruits aged 18–19 years were evaluated. High levels of SoC were directly associated with greater happiness (β = 0.49, p < 0.001), whereas higher OHIP-14 scores, indicating poorer OHRQoL, were directly associated with lower happiness (β = −0.09, p < 0.05). Untreated dental caries was indirectly associated with lower happiness (β = −0.03, p < 0.05) via OHRQoL. No significant paths were observed from socioeconomic or behavioral variables to happiness. In conclusion, psychosocial and clinical factors were associated with happiness levels among Brazilian Army recruits.

## Introduction

The perception that health is not merely the absence of disease has been widespread since the first half of the 20th century and has influenced contemporary concepts of oral health. In 2016, the FDI World Dental Federation General Assembly defined three central pillars that underpin oral health (disease state and condition, physiological function, and psychosocial function), which interact in a complex manner with other aspects, such as moderating factors, health determinants, general well-being, and overall health.^1^ Authors have reported the fundamental connections between oral health and general well-being across diverse populations and settings,^2–3^ reinforcing the need for studies investigating subjective outcomes, such as oral health-related quality of life (OHRQoL) and happiness.

According to Tatarkiewicz, happiness can be defined as "a satisfaction with life as a whole, lasting, complete and justified".^4^ OHRQoL, in turn, is a multidimensional construct that reflects the impact of oral health on individuals’ daily functions and well-being.^5^ Although a substantial body of literature has examined the association between oral health and OHRQoL, only a few studies have addressed oral health-related factors as possible predictors of happiness. Three studies have evaluated this topic in older adults^6–8^ and three others in adolescents.^9–11^ Overall, these studies reported associations between dental caries, malocclusion, socioeconomic conditions, OHRQoL, and happiness in adolescents. However, all were conducted by the same research group and involved the same sample of adolescents aged 12–14 years.^9–11^

In this context, one psychosocial attribute that may influence the relationship between oral health, OHRQoL, and happiness is the sense of coherence (SoC), which refers to the capacity to remain healthy under stressful conditions and originates from Aaron Antonovsky's salutogenic theory.^12^ Higher levels of SoC are associated with a greater ability to perceive life events as less stressful (comprehensibility), to use available resources effectively when dealing with stressors (manageability), and to perceive life as meaningful when coping with the environment (meaningfulness).^13^ This personal resource allows individuals to face stressful situations positively and maintain health.^12^

Adolescence encompasses the period from 10 to 19 years of age, during which individuals undergo important physical, emotional, and psychosocial changes that may affect oral health outcomes^14^. Early adolescence includes individuals aged 10–14 and represents the transition from childhood to adolescence, whereas late adolescence includes those aged 15–19 years and represents the transition from adolescence to adulthood. According to data from the most recent national oral health survey conducted in Brazil,^15^ the DMFT index (number of decayed, missing due to caries, or filled permanent teeth) doubles between 12 and 15–19 years of age, further justifying studies focused on this population. In addition, investigating subjective factors during this life stage is important, since psychosocial impacts arising during adolescence may have repercussions throughout the life course. From this perspective, public health interventions targeting these factors can be proposed.

To the best of our knowledge, no study has evaluated the association between oral health and happiness during late adolescence. Considering the relevance of this topic and the limited available evidence, the aim of this study was to investigate socioeconomic, psychosocial, behavioral, and clinical factors directly and indirectly associated with subjective happiness among Brazilian Army conscripts in southern Brazil through pathway analysis. This analytical approach enables the simultaneous modeling of multiple variables to estimate the complex relationships involving direct and indirect paths to the main study outcome (happiness). We hypothesized that structural determinants, such as socioeconomic conditions, influence intermediary determinants, including behavioral and clinical factors, which ultimately affect OHRQoL and, consequently, happiness. These determinants may interact, exerting both direct and indirect effects on subjective happiness.

## Methods

### Ethical aspects

The study protocol was approved by the Research Ethics Committee of the Federal University of Santa Maria (CAAE 20079591.1.0000.5346). All participants provided written informed consent, and the commanders of the involved military bases authorized the study. Recruits with treatment needs received dental treatment.

### Training and calibration

Training and calibration for dental caries examination were conducted under the supervision of a reference examiner (P.K.M.). Training included theoretical exercises based on photographs and practical training with patients not pertaining to the study sample. Subsequently, the calibration process was performed and included two stages. First, 24 adolescents not pertaining to the study sample were evaluated twice, with a 7-day interval between assessments. At this stage, intra- and interexaminer unweighted Cohen's kappa values for dental caries were 0.86 and 0.92, respectively. In the second stage, double examinations were performed in the first 12 participants of the survey, and the intra-examiner unweighted Cohen's kappa value was 0.96. The examiner was trained by an experienced periodontist to collect data on gingival bleeding; calibration was not performed, since this is a variable condition.

### Study design and sample

This cross-sectional study was conducted at two Brazilian Army military bases and included a convenience sample of conscripts drafted for mandatory military service (male adolescents aged 18–19 years). Data were collected in the city of Itaqui, Rio Grande do Sul, Brazil, between 2019 and 2020, and in Santiago, Rio Grande do Sul, Brazil, in 2021. These cities are similar in size and municipal human development index (MHDI). Itaqui has approximately 37,000 inhabitants, and Santiago has approximately 50,000 inhabitants; the MHDI values are 0.713 and 0.766, respectively^16^. Both cities have fluoridated public water supplies.

### Data collection

Clinical dental examinations were performed by a single trained and calibrated examiner (N.C.L.); all surfaces from incisors to third permanent molars were examined. The examinations followed this sequence: recording of the gingival bleeding index, professional tooth cleaning, tooth drying, and recording of the dental caries index. Gingival bleeding was recorded at four sites per tooth^17^ (distal, mesial, lingual, and buccal) and classified as absent (< 10% of bleeding sites), localized (≥ 10% to ≤ 30% of bleeding sites), or generalized gingivitis (> 30% of bleeding sites).^18^ The DMFT index was recorded to describe caries experience in the study sample. For analytical purposes, the M component was used to assess tooth loss, and the D component was used to assess untreated caries.

Three self-administered questionnaires were completed by the participants to assess the following psychosocial variables: SoC, OHRQoL, and happiness. SoC was assessed using the reduced version of the questionnaire (SoC-13) validated into Brazilian Portuguese,^19^ which consists of 13 items representing the components of comprehensibility, manageability, and meaningfulness. Responses are obtained using a five-point Likert scale, and the final score is obtained by summing all responses, ranging from 13 to 65. Higher scores indicate stronger SoC.

The OHIP-14 questionnaire,^20^ translated and cross-culturally adapted for Brazilian Portuguese,^21^ was applied to assess OHRQoL. It consists of 14 questions divided into seven domains: functional limitation, physical pain, psychological discomfort, physical disability, psychological disability, social disability, and handicap. Responses are recorded using a five-point Likert scale, where 0 indicates never, 1 is almost never, 2 is occasionally, 3 is reasonably frequent, and 4 is very often. The final score is calculated using an additive method and ranges from 0 to 56, with higher scores indicating poorer OHRQoL.

The Subjective Happiness Scale^22^ was applied to assess happiness and had been previously translated and validated for Brazilian Portuguese.^23^ It consists of four questions scored on a Likert scale from 1 to 7. For analytical purposes, the final item is reverse-coded, and the final score is calculated as the arithmetic mean of the four items, ranging from 1 to 7, where higher scores indicate greater happiness.

The recruits also completed a semi-structured questionnaire evaluating socioeconomic and behavioral factors. Family income was classified as ≤ 3 or ≥4 Brazilian minimum wages (BMW; 1 BMW = 245 USD). Maternal education and participant education were both classified as < 8 years, 8–10, or ≥ 11 years. Behavioral variables included toothbrushing frequency (≤ once/day, twice/day, or ≥ 3 times/day) and flossing (no or yes).

### Theoretical model

**Figure 1:**
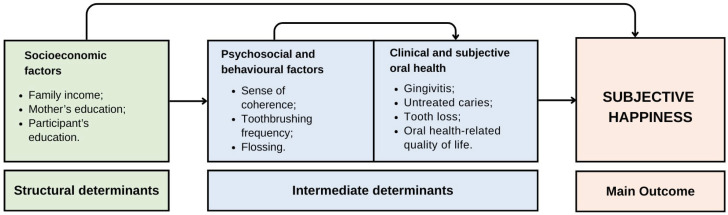
Theoretical model used to examine the relationships among socioeconomic, psychosocial, behavioral, and clinical variables and subjective happiness, adapted from the World Health Organization.^24^

The initial theoretical model was constructed according to the conceptual framework of social determinants of health^24^ and based on previous literature on this topic. Structural determinants (family income, maternal schooling, and participant education) were hypothesized to influence intermediary determinants. These include psychosocial (SoC), behavioral (toothbrushing frequency and flossing), and oral health variables, both clinical (gingivitis, untreated caries, and tooth loss) and subjective (OHRQoL), which may ultimately affect happiness. The theoretical model is shown in Figure 1.

### Data analysis

All data analyses were performed using Stata software (Stata 14.2, Stata Corporation, College Station, USA). A descriptive analysis of the main characteristics of the sample was performed. Pathway analysis was used to test direct and indirect paths linking socioeconomic, psychosocial, behavioral, and clinical factors to subjective happiness. Variables were treated as quantitative whenever possible (gingivitis, untreated caries, tooth loss, SoC, OHIP-14, and happiness). Socioeconomic and behavioral variables were treated as qualitative.

The estimation method used was maximum likelihood (ML). Model fit was evaluated using root mean square error of approximation (RMSEA), comparative fit index (CFI), Tucker–Lewis index (TLI), and standardized root mean square residual (SRMR). RMSEA values < 0.05, CFI and TLI values > 0.90, and SRMR values < 1.0 indicate adequate fit.^25^ Modification indices were used to evaluate model fit, and paths with values < 0.20 were considered non-significant and removed stepwise. Results are presented as standardized coefficients (β), 95% confidence intervals, and p-values. A significance level of 5% was adopted.

## Results

All invited recruits agreed to participate (response rate: 100%). A total of 520 participants completed the questionnaires and underwent clinical examinations; however, 21 had missing information (13 SoC, 2 OHIP-14, 1 happiness, 1 toothbrushing frequency, and 1 flossing), resulting in a final sample of 499 individuals. As shown in Table 1, most recruits belonged to families with an income of ≤ 3 BMW (75%); 43% of mothers had < 8 years of schooling, whereas 49% of participants had ≥ 11 years of education. Regarding behavioral habits, more than half of the sample reported toothbrushing ≥3 times/day, whereas 65.3% did not floss. Regarding caries prevalence, 70.7% of participants (n = 353) had DMFT ≥ 1. The mean (± standard deviation [SD]) DMFT was 2.3 (± 2.4), ranging from 0 to 14. The mean numbers of untreated caries lesions and missing teeth were both 0.4. Most participants had gingivitis, either localized or generalized, and the mean gingival bleeding index was 21%. On average, the happiness score was 5.2, ranging from 1.5 (least happy) to 7 (happiest). Additional descriptive data are presented in Table 1.

**Table 1:** Sample characteristics.

Qualitative variables			n (%)	95% CI		
Socioeconomic variables						
	Family income					
		≤ 3 BMW	374 (75.0)	71.0–78.8		
		≥ 4 BMW	125 (25.0)	21.4–29.0		
	Mother's education					
		< 8 years	215 (43.1)	38.8–47.5		
		8–10 years	96 (19.2)	16.0–22.9		
		≥ 11 years	188 (37.7)	33.5–42.0		
	Participant education					
		< 8 years	73 (14.6)	11.8–18.0		
		8–10 years	181 (36.3)	32.1–40.6		
		≥ 11 years	245 (49.1)	44.7–53.5		
Behavioral variables						
	Toothbrushing frequency					
		≤ once/day	29 (5.8)	4.1–8.3		
		Twice/day	190 (38.1)	33.9–42.4		
		≥ 3 times/day	280 (56.1)	51.7–60.4		
	Flossing					
		No	326 (65.3)	61.0–69.4		
		Yes	173 (34.7)	30.6–39.0		
Clinical variables						
	Caries prevalence					
		DMFT = 0	146 (29.3)	25.4–33.4		
		DMFT ≥ 1	353 (70.7)	66.6–74.6		
	Tooth loss prevalence					
		MT = 0	391 (78.4)	74.5–81.8		
		MT ≥ 1	108 (21.6)	18.2–25.5		
	Gingivitis					
		Absent	158 (31.7)	27.7–35.9		
		Localized	212 (42.5)	38.2–46.9		
		Generalized	129 (25.8)	22.2–29.9		
**Quantitative variables**			**Mean (SD)**	**95% CI**	**Median (IQR)**	**Range**
Clinical variables						
	DMFT		2.3 (2.4)	2.1–2.5	2 (0, 4)	0–14
	Untreated caries (DT)		0.4 (0.8)	0.3–0.5	0 (0, 1)	0–5
	Missing teeth (MT)		0.4 (0.9)	0.3–0.5	0 (0, 0)	0–4
	Gingival bleeding index		0.21 (0.18)	0.20–0.23	0.16 (0.08, 0.30)	0–0.95
Psychosocial variables						
	OHRQoL		9.1 (7.2)	8.5–9.8	8 (4, 13)	0–38
	SoC		46.5 (7.1)	45.9–47.2	47 (42, 52)	24–63
	Happiness		5.2 (1.2)	5.15–5.35	5.5 (4.5, 6.25)	1.5–7

BMW: Brazilian minimum wage; DMFT: number of decayed: missing due to caries, or filled permanent teeth; MT: number of missing permanent teeth; DT: number of decayed permanent teeth; SD: standard deviation; CI: confidence interval; IQR: interquartile range; OHRQoL: oral health–related quality of life; SoC: sense of coherence.

**Figure 2:**
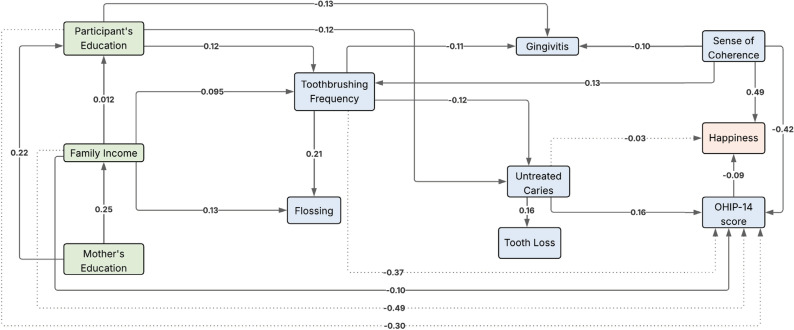
Significant associations identified by pathway analysis linking explanatory variables and happiness (p < 0.05). Solid lines indicate direct effects, and dashed lines indicate indirect effects.

Figure 2 illustrates the direct and indirect paths linking socioeconomic, psychosocial, behavioral, and clinical variables to happiness. Higher SoC levels were directly associated with greater happiness (β = 0.49, p < 0.001). In addition, greater OHRQoL impacts were directly associated with lower happiness (β = −0.09, p < 0.05). Untreated dental caries indirectly affected happiness levels via OHRQoL (β = −0.03, p < 0.05). No significant paths were observed from socioeconomic or behavioral variables to happiness. Standardized effects between predictor variables and happiness in the initial and final structural models are presented in Table 2. The parsimonious model demonstrated good fit, as evidenced by RMSEA (0.03; 0.00–0.05), CFI (0.98), and TLI (0.948).

**Table 2:** Standardized effects among the predictor variables and subjective happiness in the initial and final structural model.

Direct pathway to		Initial model		Final model	
		² (95%CI)	p-value	² (95%CI)	p-value
Subjective happiness					
	Sense of coherence	0.49 (0.42 to 0.57)	<0.001	0.49 (0.42 to 0.57)	<0.001
	Gingivitis	0.07 (-0.01 to 0.14)	0.09	0.07 (-0.01 to 0.14)	0.09
	Untreated caries	0.01 (-0.07 to 0.84)	0.86	0.01 (-0.07 to 0.08)	0.86
	Tooth loss	−0.04 (-0.12 to 0.03)	0.24	−0.04 (-0.12 to 0.03)	0.24
	OHIP-14	−0.09 (-0.17 to −0.004)	0.04	−0.09 (-0.17 to −0.01)	0.04
OHIP-14					
	Family income	−0.09 (-0.17 to −0.01)	0.02	−0.10 (-0.17 to −0.02)	0.02
	Mother's education	−0.02 (-0.10 to 0.06)	0.67	−	
	Participant's education	−0.06 (-0.14 to 0.02)	0.15	−0.06 (-0.14 to 0.02)	0.12
	Sense of coherence	−0.42 (-0.50 to −0.35)	<0.001	−0.42 (-0.50 to −0.35)	<0.001
	Gingivitis	0.05 (-0.02 to 0.13)	0.17	0.05 (-0.02 to 0.13)	0.17
	Untreated caries	0.16 (0.08 to 0.23)	<0.001	0.16 (0.08 to 0.24)	< 0.001
	Tooth loss	0.04 (-0.04 to 0.12)	0.30	0.04 (-0.04 to 0.12)	0.30
Gingivitis					
	Family income	−0.01 (-0.10 to 0.08)	0.84	−	
	Mother's education	−0.06 (-0.10 to 0.08)	0.90	−	
	Participant's education	−0.13 (-0.22 to −0.04)	0.004	−0.13 (-0.22 to −0.05)	0.002
	Sense of coherence	−0.10 (-0.19 to −0.02)	0.02	−0.10 (-0.19 to −0.02)	0.02
	Toothbrushing frequency	−0.11 (-0.20 to −0.02)	0.02	−0.11 (-0.20 to −0.02)	0.02
	Flossing	−0.06 (-0.15 to 0.02)	0.16	−0.06 (-0.15 to 0.02)	0.15
Untreated caries					
	Family income	−0.07 (-0.16 to 0.02)	0.12	−0.07 (-0.16 to 0.02)	0.12
	Participant's education	−0.12 (-0.21 to −0.03)	0.008	−0.12 (-0.21 to −0.03)	0.008
	Sense of coherence	−0.01 (-0.09 to 0.08)	0.87	−	
	Toothbrushing frequency	−0.12 (-0.21 to −0.03)	0.006	−0.12 (-0.21 to −0.04)	0.006
	Flossing	−0.02 (-0.11 to 0.07)	0.68	−0.02 (-0.11 to 0.07)	0.67
Tooth loss					
	Family income	−0.06 (-0.15 to 0.02)	0.15	−0.06 (-0.15 to 0.02)	0.15
	Participant's education	0.01 (-0.09 to 0.09)	0.99	−	
	Sense of coherence	0.05 (-0.04 to 0.14)	0.26	0.05 (-0.04 to 0.14)	0.26
	Toothbrushing frequency	−0.07 (-0.16 to 0.02)	0.12	−0.07 (-0.16 to 0.02)	0.12
	Untreated caries	0.16 (0.07 to 0.25)	<0.001	0.16 (0.07 to 0.25)	< 0.001
Toothbrushing frequency					
	Family income	0.09 (0.01 to 0.18)	0.003	0.095 (0.01 to 0.18)	0.03
	Mother's education	0.06 (-0.03 to 0.15)	0.21	0.06 (-0.03 to 0.15)	0.21
	Participant's education	0.12 (0.04 to 0.21)	0.006	0.12 (0.03 to 0.21)	0.006
	Sense of coherence	0.13 (0.04 to 0.21)	0.003	0.13 (0.04 to 0.21)	0.003
Flossing					
	Family income	0.13 (0.04 to 0.21)	0.004	0.13 (0.04 to 0.21)	0.003
	Mother's education	−0.01 (-0.10 to 0.08)	0.84	−	
	Participant's education	0.03 (-0.06 to 0.11)	0.55	−	
	Sense of coherence	0.07 (-0.02 to 0.15)	0.11	0.07 (-0.02 to 0.15)	0.11
	Toothbrushing frequency	0.21 (0.12 to 0.29)	<0.001	0.21 (0.13 to 0.29)	< 0.001
Sense of coherence					
	Family income	0.08 (-0.01 to 0.17)	0.10	−	
	Mother's education	−0.10 (-0.19 to −0.01)	0.03	−0.08 (-0.17 to 0.01)	0.06
	Participant's education	−0.01 (-0.09 to 0.09)	0.99	−	
	Participant's education				
	Family income	0.12 (0.04 to 0.21)	0.004	0.12 (0.04 to 0.21)	0.004
	Mother's education	0.22 (0.13 to 0.30)	<0.001	0.22 (0.13 to 0.30)	<0.001
Family income					
	Mother's education	0.25 (0.16 to 0.33)	<0.001	0.25 (0.16 to 0.33)	<0.001
**Indirect** * **Pathway from**		**Initial model**		**Final model**	**p-value**
		**β (SE)**	**p-value**	**β (SE)**	
Untreated caries to subjective happiness					
	Via OHIP-14	−0.03 (0.01)	0.03	−0.03 (0.01)	0.03
Model Fit					
	RMSEA (90% CI)	0.05 (0.03 to 0.08)		0.03 (0.00 to 0.05)	
	CFI	0.969		0.980	
	TLI	0.859		0.948	

²: beta coefficient; SE: standard error; OHIP: Oral Health Impact Profile; RMSEA: Root Mean Square Error of Approximation; CI: Confidence interval; CFI: Comparative Fit Index; TLI: Tucker-Lewis Index;

* only significant indirect pathways related to subjective happiness.

Sample size adequacy was verified by power analysis for pathway analysis. Considering an alpha error of 0.05, 55 degrees of freedom, a sample size of 499 individuals, and initial and final RMSEA values of 0.051 and 0.031, respectively, the estimated study power was 82%.

## Discussion

This study investigated the direct and indirect paths linking socioeconomic, psychosocial, behavioral, and clinical factors to subjective happiness among recruits from two military bases in southern Brazil. Based on the findings, direct paths were identified between psychosocial variables (SoC and OHRQoL) and happiness. Regarding clinical variables, an indirect path was observed between the number of untreated dental caries and happiness via OHRQoL. To the best of our knowledge, this is the first study to investigate the association between oral health-related factors and happiness using pathway analysis in a sample of late adolescents.

As research advances, increasing attention has been directed toward psychosocial aspects and their relationship with health and well-being, including specific nuances of the stomatognathic system. Current perceptions underscore the influence that social and psychological attributes, together with their adaptive capabilities, exert on oral health^1^. In this context, the association between SoC and oral health outcomes has been documented in the literature. Previous systematic reviews have reported that high levels of SoC act both as a protective factor against risky health behaviors and as a promoter of healthy attitudes.^26–28^ In the present study, in addition to a direct path linking SoC to OHRQoL (an association previously demonstrated in a study conducted by our research group using a different statistical approach),^29^ a direct path linking stronger SoC to greater happiness was also observed. This finding may be explained by the fact that a strong SoC indicates an individual who has effective tension-management capabilities, reflects on available resources, and can identify and mobilize them, thereby enabling effective coping and problem solving, which may ultimately influence happiness and life satisfaction.^12^

Our findings also demonstrated that individuals experiencing greater impacts on OHRQoL reported lower happiness, through a direct association. This result highlights the influence of oral health conditions on adolescents’ happiness. These findings are consistent with those of Tuchtenhagen et al.,^9^ who reported that poorer OHRQoL was associated with lower happiness among 12-year-old adolescents from southern Brazil in both a cross-sectional study and a cohort study that followed the same sample over two years.^11^ Thus, individuals who perceive greater oral health impacts on quality of life—such as pain, eating or sleeping limitations, emotional distress, or difficulties in social interaction—are directly affected in terms of their happiness, reinforcing the present findings.^1,9,11^

Regarding clinical variables, an indirect path linking a greater number of decayed teeth to lower levels of happiness via OHRQoL was identified. The relationship may be explained by the increased likelihood of dental pain, chewing difficulties, and other impairments associated with untreated caries, which negatively affect OHRQoL and, consequently, happiness.^30^ A negative association between untreated caries and happiness was also reported by Tuchtenhagen et al. in both cross-sectional^9^ and cohort^11^ studies involving adolescents from southern Brazil. However, those studies did not use pathway analysis to examine multiple interrelated pathways among variables. Because the overall OHIP-14 score was used in the present analytical approach, it was not possible to identify which specific domains were associated with lower happiness.

In the present study, no significant paths were observed linking socioeconomic or behavioral variables to happiness. In contrast, Tuchtenhagen et al.,^11^ using a different analytical approach, found that adolescents from lower-income families, with less-educated mothers, and living in more crowded households reported lower happiness. In the current sample, however, socioeconomic variables (family income and participant education) were directly associated with behavioral variables (toothbrushing and flossing) and/or clinical variables (gingivitis and untreated caries). One possible explanation for the lack of a direct association between socioeconomic variables and happiness in this study is that some variables were collected in categorical form, which limited their inclusion as numerical indicators in the pathway analysis.

This study has some limitations that must be acknowledged, including its cross-sectional design, which precludes causal inference, particularly in the context of pathway analysis. In addition, OHRQoL, SoC, and happiness were treated as observed variables, although they could also be modeled as latent constructs. Although validated instruments were used, modeling these constructs as latent variables could provide more precise estimates by accounting for measurement error. Another limitation relates to the study sample, which consisted of a convenience sample of recruits drafted for mandatory military service. Therefore, the findings cannot be extrapolated to adolescents in general, including females or younger adolescents. Nevertheless, the homogeneity of the sample in terms of age and sex may be considered a strength, since it reduces potential confounding. Furthermore, although this study investigated some psychosocial factors associated with happiness, other relevant characteristics, such as social capital and social cohesion, were not assessed. Future studies may examine a broader range of psychosocial variables and their relationship with happiness during adolescence. Finally, the use of ML estimation may also be considered a limitation, since it is less optimal for ordinal variables compared with WLSMV (weighted least squares mean and variance adjusted). However, most variables were continuous, and the ordinal measures followed an approximately continuous distribution, supporting the use of ML in this context. In addition, although some coefficients were small, indicating modest effects, the model was adequate for the study aims and yielded stable and interpretable results.

This study also has notable strengths, including the use of pathway analysis, which enables assessment of a network of relationships among multiple factors that may ultimately affect happiness. Another strength lies in the investigation of subjective aspects related to oral health in diverse populations, particularly in specific groups that have been insufficiently studied. In this context, the main outcome of the present study—happiness—deserves emphasis, since it is a subjective and complex construct that has been rarely investigated, despite its relevance to health, as highlighted in an editorial published in *The Lancet* in 2016.^31^ Furthermore, this study examined important factors during a critical period of life, which may have repercussions throughout the life course. Thus, the findings may contribute to the development of public health policies and oral health promotion strategies emphasizing psychosocial factors, which appear to be particularly relevant during adolescence. Future studies using longitudinal designs and intervention approaches are warranted to further explore these relationships.

In conclusion, this study demonstrated that psychosocial and clinical factors were associated with happiness levels among Brazilian Army recruits from southern Brazil. These findings may inform public health policies and oral health promotion programs aimed at strengthening SoC and improving oral health, which may ultimately promote happiness during adolescence.

## Data Availability

The datasets generated during and/or analyzed during the current study are available from the corresponding author on reasonable request.
